# Supplementary material for: Multi-omics Visualization Platform: An extensible Galaxy plug-in for multi-omics data visualization and exploration
Source: Gigascience. 2020 Mar 28;9(4):giaa025. doi: 10.1093/gigascience/giaa025 (PMC7102281; doi:10.1093/gigascience/giaa025)
Supplement: giaa025_Supplemental_Files [file giaa025_supplemental_files.zip › AdditionaLFile_1.pdf]

Additional File 1

Example visualization of novel splice junction peptide

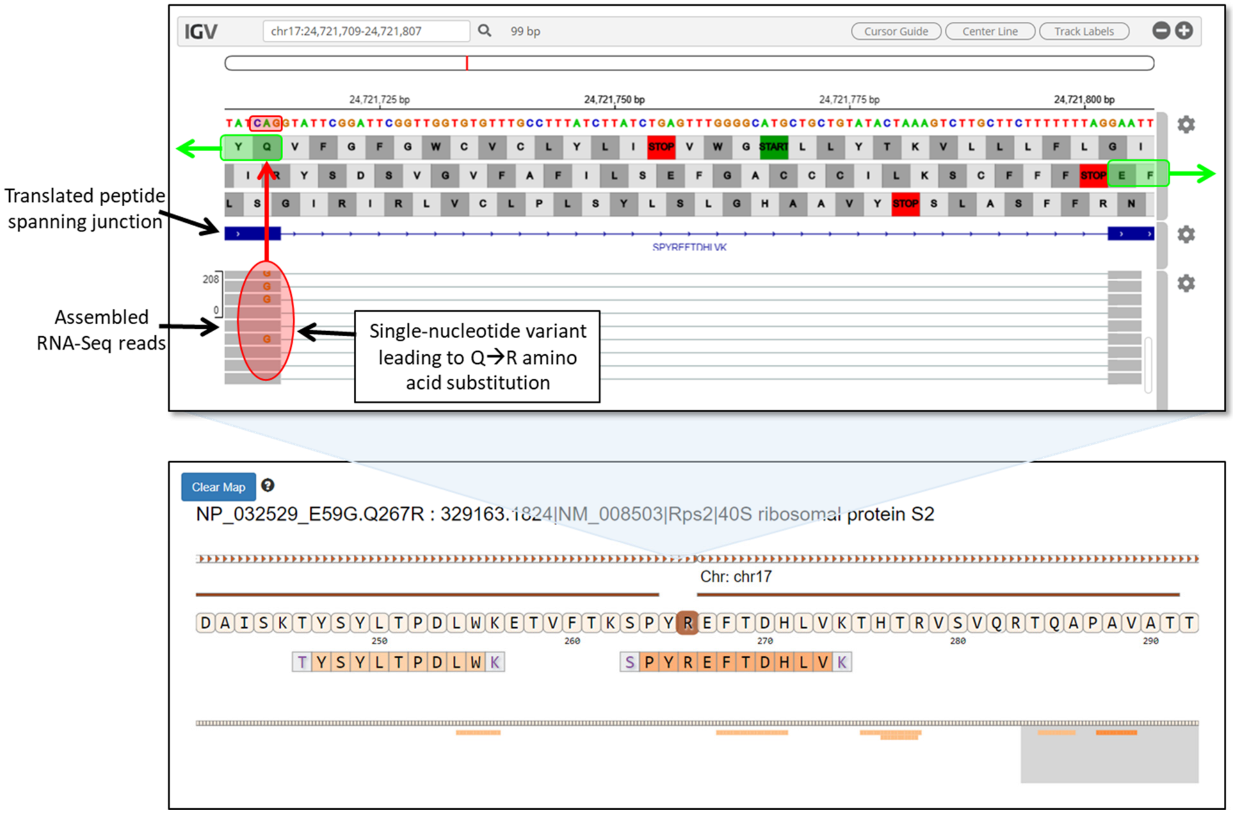

Lower panel. The figure shows a potential novel peptide sequence identified and visualized in the Peptide-Protein Viewer. The peptide contains a single amino acid substitution at position 267, substituting an R (in dark shading) for the reference Q amino acid at this position.

Upper panel. Opening up the IGV viewer from the Peptide-Protein Viewer shows that this peptide not only contains a single amino acid substitution, but also crossing a novel splice junction. The RNA-Seq reads indicated a G amino acid within the positive (sense) DNA strand (red shaded circle), indicating a codon sequence change from CAG to CGG in the genomic sequence, and leading to the substitution of R for Q at this position. The green shaded boxes show the amino acids within the identified peptide SP**YR-EF**TDHLVK which span a novel splice junction and are aligned with the RNA-Seq reads shown in gray in the lower track. The amino acids joined together across the junction and shown in the upper panel are in bold (--YREF--).
