# Supplementary material for: Multi-omics Visualization Platform: An extensible Galaxy plug-in for multi-omics data visualization and exploration
Source: Gigascience. 2020 Mar 28;9(4):giaa025. doi: 10.1093/gigascience/giaa025 (PMC7102281; doi:10.1093/gigascience/giaa025)
Supplement: giaa025_Supplemental_Files [file giaa025_supplemental_files.zip › AdditionalFile_2.pdf]

Additional File 2-- Schema of databases and tables acting as input to MVP

1) mzSQLite database schema

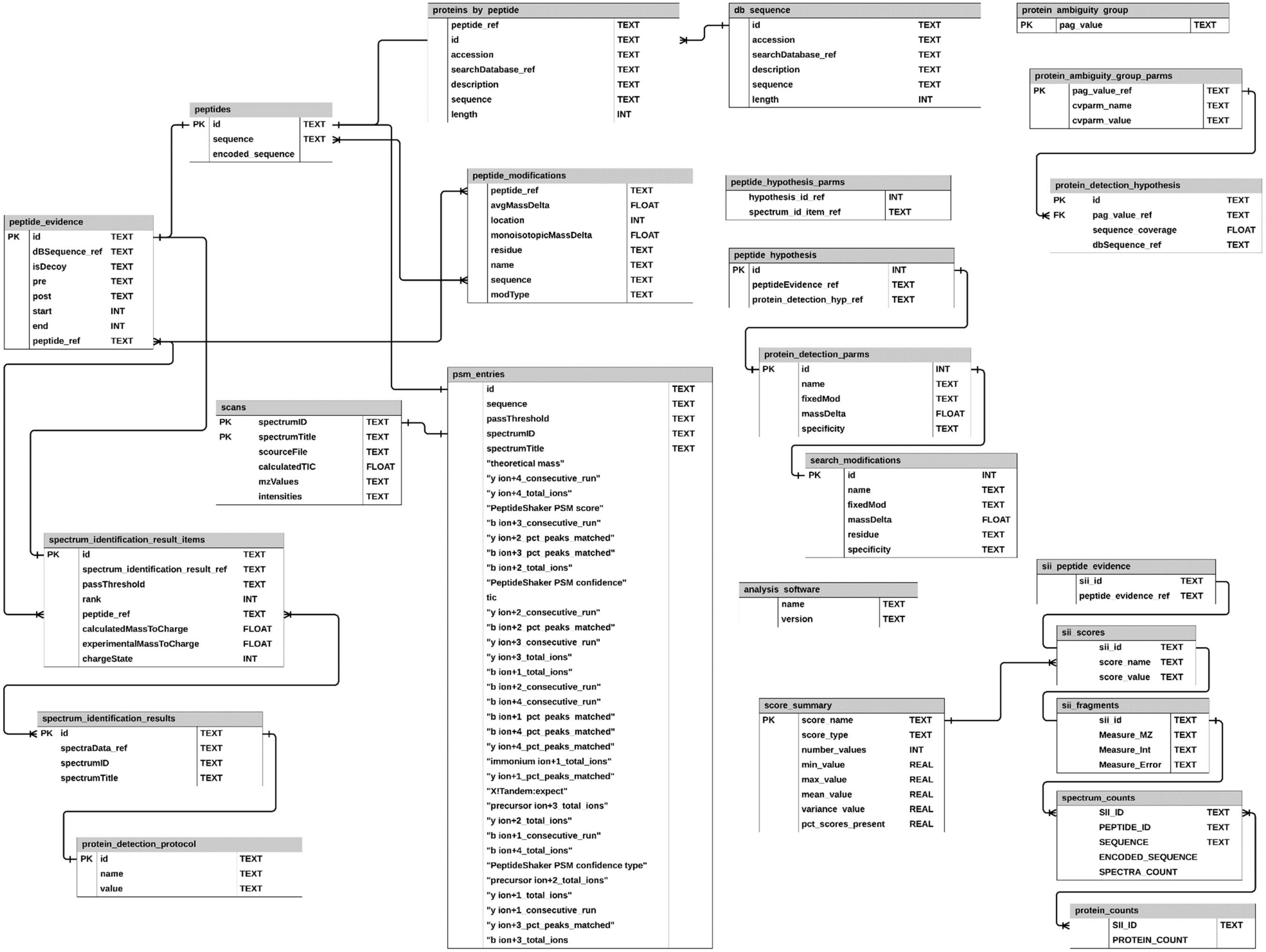

2) variant_annotation table and the feature_cds_map table schema

feature_cds_map		
	name	TEXT
	chrom	TEXT
	start	INT
	end	INT
	strand	TEXT
	cds_start	INT
	cds_end	INT

variant_annotation		
	name	TEXT
	reference	TEXT
	cigar	INT
	annotation	INT
