# Supplementary material for: Multi-omics Visualization Platform: An extensible Galaxy plug-in for multi-omics data visualization and exploration
Source: Gigascience. 2020 Mar 28;9(4):giaa025. doi: 10.1093/gigascience/giaa025 (PMC7102281; doi:10.1093/gigascience/giaa025)
Supplement: giaa025_Response_to_Reviewer_Comments_Original_Submission [file giaa025_response_to_reviewer_comments_original_submission.pdf]

Author's Response To Reviewer Comments

Close

Point-by-point response to reviewers

Reviewer #1: The manuscript describes a Galaxy plug-in, MVP, that allows users to visualize annotated MS/MS of certain peptides for validation or filtering purpose in proteogenomics. If a peptide harbors mutations, the tool also provides an IGV view of genomic and transcriptomic information underlying this peptide. It is a useful tool in my view. The manuscript is well written.

>We thank the reviewer for the positive comments on our described work.

1. Although the manuscript is focused on MVP, the input files are heavily reliant on the previous steps that may involve a number of proteogenomics tools (Figure 1). It is not an issue for an experienced proteomics researcher, but it could be confusing for someone who is less informed of the field. Thus I think a brief description of the process would be very helpful , including which tool generate each input files.

>This is an excellent suggestion. In response, we have added a paragraph immediately preceding Figure 1 in the text which describes available resources (workflows and documentation) for the upstream workflows which generate data that ultimately is used as inputs to MVP. Training documentation is available on the Galaxy Training Network online resource for these workflows. We have also added the links to these training resources in the Accessibility section.

2. For a better understanding of the data structure in the variant_annotation table, I would suggest adding one example for each type of variant (single amino acid change, deletion, insertion etc.).

>Thank you for this suggestion. We have expanded Table 1, now including examples of frameshifts due to single nucleotide insertion/deletion, short Indels and structural variants (fusion), and how these are depicted in the variant annotation table. We have also clarified that MVP reads only the “name TEXT” and “cigar TEXT” fields for displaying variant sequences. The other fields are descriptive information included in the table for completeness.

3. In table 2, the authors highlight the capability of the annotation table to represent gene fusion cases. I suggest them to include examples in the table to make the function more intuitive.

>Thank you for this suggestion. We have expanded Table 2, showing an example depiction of a fusion variant (the same fusion detailed in the variant_annotation examples in Table 1). The fusion is depicted as two entries in the feature_cds_map table -- one entry for each of the two chromosomal regions fused together to form the fusion gene and expressed gene products.

4. Please check all the links in the manuscript. Several of them seem not working. (page 26 for example)

>We have checked all links in the manuscript, including those listed in References cited. They are all working at the time of this submission.

Reviewer #2: Authors of the paper have put together an excellent visualization tool in Galaxy to visualize and analyze Protegenomics data.

The tool's source code is available from GitHub and the visualization plugin is also available from usegalaxy.eu which proves that authors are trying to make the tool open and accessible to many users. I have tested the tool on usegalaxy.eu, and it lives up to the functionalities mentioned in the

manuscript.

>We thank the reviewer for the positive comments on our described work.

However, I think the manuscript is not justifying the purpose and functionality of the tool correctly, so I advise the following suggestions to improve the manuscript.

1. Update legend for figure 1 mentioning the process shown in the figure or if the process is not essential then update figure just showing inputs.

>This is an excellent suggestion, and in response we have added a paragraph in the text describing the processes shown in Figure 1. Specifically, we have added text which details the upstream process for generating the protein FASTA sequence database from RNA-Seq data and also the process for matching MS/MS data to peptide sequences using this database. These are important upstream workflows which ultimately generate results which provide inputs for MVP. Importantly, we have developed accessible workflows and training documentation enabling interested researchers to generate the results which are used as inputs for MVP. We provide readers information in the Accessibility section on accessing these workflows and documentation through the online Galaxy Training Network.

2. As it is a technical paper, provide a database schema for input(s).

>We thank the reviewer for this suggestion. Including the schema will provide a more complete description of the inputs to MVP. We have added Additional File 2, which provides Entity Relationship diagrams for the schema for the databases and tables which act as input to MVP: the mzSQLite database, the variant_annotation table and the feature_cds_map table.

3. variant_annotation and feature_cds_map are sqlite tables in the databases with same name which could be confusing, change the text accordingly to make it clear.

>In the Operation section of the text, we made revisions to clarify that the variant_annotation and feature_CDS_map are separate SQLite data tables, which are not contained in the same database. These are generated within the Galaxy History and used as data inputs to MVP. Because these are separate data tables, naming of columns etc. that may overlap between the tables does not create issues in MVP.

4. Page 9, line 1 "For each exon coding a protein,.." do you mean "For each coding exons of a protein"? Please clarify.

>We have changed the text as suggested by the reviewer to read: "For each coding exon of a translated protein…"

5. Page 10, line 7, the authors mention that plugin interacts with other input data types if present, does the plugin read these data files directly or needed to load manually, if it loads automatically is it based on naming convention? If possible provide an example in functionality section.

>In the Operation section, where we describe Figure 2 and the invocation of MVP from the Galaxy History, we have added text clarifying that MVP automatically accesses compatible inputs such as the variant_annotation and feature_cds_map tables through functionalities within MVP which read these specific inputs. We point the readers to the Functionality section of the manuscript where we further describe these functions and how these features automatically access these inputs from the active Galaxy history (see description of Figure 5, parts C and D).

6. Page 12, line 4, provide a direct link to sample dataset.

>Rather than provide links in this section of the text to the demonstration datasets and workflows, we point the reader to the Accessibility section of the manuscript where instructions and links are provided to access data and software.

7. Page 12, line 12, change figure reference to correct figure.

>We have changed this reference to Figure 2 and not Figure 1.

8. Page 14, line 21, change the text 'see below' to the appropriate figure/section.

>We have added text pointing the reader to the specific Figure in the text below that describes the functionalities in more detail.

9. Page 16, line 2-6, may be move Lorikeet introduction to Findings.

>We have made revisions as suggested. Lorikeet is first introduced in the manuscript in the Operation section of the Findings, where a reference is provided with more information on the tool, and the purpose for its use is described. In response to comment #10 below, we have revised the description of Lorikeet in the Functionality section to give a more detailed description of its features.

10. Page 16, line 2 introduces Lorikeet viewer it seems like an informative tool but not described what it is, so please briefly describe what the user is looking at in figure 4c.

>This is an excellent suggestion, and we have provided more detail in the text which describes the data viewed in Figure 4C.

11. Figure 5 legend, describe what other colors like grey amino acids either side and, different shades of orange (not in this figure, but I noticed in the demo).

>We have added additional text describing color schemes in the protein map (5A) and the zoomed-in visualization of specific peptide sequences (5B).

12. Page 19, line 1, part D in figure 4 is not present; do you mean figure 5?

>This has been corrected. We did mean to refer to part D of Figure 5.

13. Page 21, line 6, Mentioned Java, do you mean JavaScript?

>This has been corrected, and now reads “JavaScript”.

14. Page 21, line 12 Author mentioned "our recently developed tool" Please provide a list and/or reference.

>We have revised and clarified - we are describing a published Galaxy tool which further assesses functional impact of sequence variants, described in Reference 18.

Close
