# Supplementary material for: Multi-omics Visualization Platform: An extensible Galaxy plug-in for multi-omics data visualization and exploration
Source: Gigascience. 2020 Mar 28;9(4):giaa025. doi: 10.1093/gigascience/giaa025 (PMC7102281; doi:10.1093/gigascience/giaa025)
Supplement: giaa025_Reviewer_1_Report_Original_Submission — Xiaojing Wang -- 12/19/2019 Reviewed [file giaa025_reviewer_1_report_original_submission.pdf]

Reviewer Report

Title: Multi-omics Visualization Platform: An extensible Galaxy plug-in for multi-omics data visualization and exploration

Version: Original Submission **Date:** 12/19/2019

Reviewer name: Xiaojing Wang

Reviewer Comments to Author:

The manuscript describes a Galaxy plug-in, MVP, that allows users to visualize annotated MS/MS of certain peptides for validation or filtering purpose in proteogenomics. If a peptide harbors mutations, the tool also provides an IGV view of genomic and transcriptomic information underlying this peptide. It is a useful tool in my view. The manuscript is well written.

1. Although the manuscript is focused on MVP, the input files are heavily reliant on the previous steps that may involve a number of proteogenomics tools (Figure 1). It is not an issue for an experienced proteomics researcher, but it could be confusing for someone who is less informed of the field. Thus I think a brief description of the process would be very helpful , including which tool generate each input files.
2. For a better understanding of the data structure in the variant_annotation table, I would suggest adding one example for each type of variant (single amino acid change, deletion, insertion etc.).
3. In table 2, the authors highlight the capability of the annotation table to represent gene fusion cases. I suggest them to include examples in the table to make the function more intuitive.
4. Please check all the links in the manuscript. Several of them seem not working. (page 26 for example)

Level of Interest

Please indicate how interesting you found the manuscript: Choose an item.

Quality of Written English

Please indicate the quality of language in the manuscript: Choose an item.

Declaration of Competing Interests

Please complete a declaration of competing interests, considering the following questions:

- Have you in the past five years received reimbursements, fees, funding, or salary from an organisation that may in any way gain or lose financially from the publication of this manuscript, either now or in the future?

- Do you hold any stocks or shares in an organisation that may in any way gain or lose financially from the publication of this manuscript, either now or in the future?
- Do you hold or are you currently applying for any patents relating to the content of the manuscript?
- Have you received reimbursements, fees, funding, or salary from an organization that holds or has applied for patents relating to the content of the manuscript?
- Do you have any other financial competing interests?
- Do you have any non-financial competing interests in relation to this paper?

If you can answer no to all of the above, write 'I declare that I have no competing interests' below. If your reply is yes to any, please give details below.

I declare that I have no competing interests.

I agree to the open peer review policy of the journal. I understand that my name will be included on my report to the authors and, if the manuscript is accepted for publication, my named report including any attachments I upload will be posted on the website along with the authors' responses. I agree for my report to be made available under an Open Access Creative Commons CC-BY license (http://creativecommons.org/licenses/by/4.0/). I understand that any comments which I do not wish to be included in my named report can be included as confidential comments to the editors, which will not be published.

Choose an item.

To further support our reviewers, we have joined with Publons, where you can gain additional credit to further highlight your hard work (see: https://publons.com/journal/530/gigascience). On publication of this paper, your review will be automatically added to Publons, you can then choose whether or not to claim your Publons credit. I understand this statement.

Yes Choose an item.
