# Supplementary material for: Multi-omics Visualization Platform: An extensible Galaxy plug-in for multi-omics data visualization and exploration
Source: Gigascience. 2020 Mar 28;9(4):giaa025. doi: 10.1093/gigascience/giaa025 (PMC7102281; doi:10.1093/gigascience/giaa025)
Supplement: giaa025_Reviewer_2_Report_Original_Submission — Anil Thanki -- 1/6/2020 Reviewed [file giaa025_reviewer_2_report_original_submission.pdf]

Reviewer Report

Title: Multi-omics Visualization Platform: An extensible Galaxy plug-in for multi-omics data visualization and exploration

Version: Original Submission **Date:** 1/6/2020

Reviewer name: Anil Thanki

Reviewer Comments to Author:

Authors of the paper have put together an excellent visualization tool in Galaxy to visualize and analyze Protegenomics data.
The tool's source code is available from GitHub and the visualization plugin is also available from usegalaxy.eu which proves that authors are trying to make the tool open and accessible to many users. I have tested the tool on usegalaxy.eu, and it lives up to the functionalities mentioned in the manuscript. However, I think the manuscript is not justifying the purpose and functionality of the tool correctly, so I advise the following suggestions to improve the manuscript.
()
1. Update legend for figure 1 mentioning the process shown in the figure or if the process is not essential then update figure just showing inputs.
2. As it is a technical paper, provide a database schema for input(s).
3. variant_annotation and feature_cds_map are sqlite tables in the databases with same name which could be confusing, change the text accordingly to make it clear.
4. Page 9, line 1 "For each exon coding a protein,.." do you mean "For each coding exons of a protein"? Please clarify.
5. Page 10, line 7, the authors mention that plugin interacts with other input data types if present, does the plugin read these data files directly or needed to load manually, if it loads automatically is it based on naming convention? If possible provide an example in functionality section.
6. Page 12, line 4, provide a direct link to sample dataset.
7. Page 12, line 12, change figure reference to correct figure.
8. Page 14, line 21, change the text 'see below' to the appropriate figure/section.
9. Page 16, line 2-6, may be move Lorikeet introduction to Findings.
10. Page 16, line 2 introduces Lorikeet viewer it seems like an informative tool but not described what it is, so please briefly describe what the user is looking at in figure 4c.
11. Figure 5 legend, describe what other colors like grey amino acids either side and, different shades of orange (not in this figure, but I noticed in the demo).
12. Page 19, line 1, part D in figure 4 is not present; do you mean figure 5?
13. Page 21, line 6, Mentioned Java, do you mean JavaScript?
14. Page 21, line 12 Author mentioned "our recently developed tool" Please provide a list and/or reference.
Overall, the authors have put together a helpful tool and a manuscript describing its operations and

functionalities. With the above corrections, I would like to see this manuscript published.

Level of Interest

Please indicate how interesting you found the manuscript: Choose an item.

Quality of Written English

Please indicate the quality of language in the manuscript: Choose an item.

Declaration of Competing Interests

Please complete a declaration of competing interests, considering the following questions:

- Have you in the past five years received reimbursements, fees, funding, or salary from an organisation that may in any way gain or lose financially from the publication of this manuscript, either now or in the future?
- Do you hold any stocks or shares in an organisation that may in any way gain or lose financially from the publication of this manuscript, either now or in the future?
- Do you hold or are you currently applying for any patents relating to the content of the manuscript?
- Have you received reimbursements, fees, funding, or salary from an organization that holds or has applied for patents relating to the content of the manuscript?
- Do you have any other financial competing interests?
- Do you have any non-financial competing interests in relation to this paper?

If you can answer no to all of the above, write 'I declare that I have no competing interests' below. If your reply is yes to any, please give details below.

I declare that I have no competing interests

I agree to the open peer review policy of the journal. I understand that my name will be included on my report to the authors and, if the manuscript is accepted for publication, my named report including any attachments I upload will be posted on the website along with the authors' responses. I agree for my report to be made available under an Open Access Creative Commons CC-BY license (http://creativecommons.org/licenses/by/4.0/). I understand that any comments which I do not wish to be included in my named report can be included as confidential comments to the editors, which will not be published.

Choose an item.

To further support our reviewers, we have joined with Publons, where you can gain additional credit to further highlight your hard work (see: https://publons.com/journal/530/gigascience). On publication of this paper, your review will be automatically added to Publons, you can then choose whether or not to claim your Publons credit. I understand this statement.

Yes Choose an item.
